# High-Performance Transparent PEDOT: PSS/CNT Films for OLEDs

**DOI:** 10.3390/nano11082067

**Published:** 2021-08-15

**Authors:** Ying Tian, Tao Wang, Qingxia Zhu, Xingcai Zhang, Anita Sagadevan Ethiraj, Wen-Ming Geng, Hong-Zhang Geng

**Affiliations:** 1 Tianjin Key Laboratory of Advanced Fibers and Energy Storage, State Key Laboratory of Separation Membranes and Membrane Processes, School of Material Science and Engineering, Tiangong University, Tianjin 300387, China; y_tian123@163.com (Y.T.); wangtao145411@foxmail.com (T.W.); 2 John A. Paulson School of Engineering and Applied Sciences, Harvard University, Cambridge, MA 02138, USA; xingcai@mit.edu; 3 Department of Physics, VIT-AP University, Amaravati 522237, Andhra Pradesh, India; ethiraj_anita25@yahoo.com; 4 Carbon Star Technology (Tianjin) Co., Ltd., Tianjin 300382, China; gengwm863@163.com

**Keywords:** carbon nanotubes, PEDOT: PSS, spin coating, OLEDs, lifetime

## Abstract

Improved OLED systems have great potential for next-generation display applications. Carbon nanotubes (CNTs) and the conductive polymers poly (3,4-ethylenedioxythiophene): poly (styrene sulfonate) (PEDOT: PSS) have attracted great interest for advanced applications, such as optoelectronic products. In this paper, the simultaneous enhancement of the conductivity, roughness, and adhesion properties of transparent conductive films with PEDOT: PSS/CNTs is reported. These films prepared by a simple spin-coating process were successfully used to produce high-performance organic light-emitting diodes (OLEDs) with an improved lifetime. Addition of PEDOT: PSS lowered the film sheet resistance and CNTs helped to enhance the stability and maintain the lifetime of the OLEDs. In addition, treatment with methanol and nitric acid changed the morphology of the polymer film, which led to greatly reduced sheet resistance, enhanced substrate adhesion, and reduced film roughness. The best performance of the film (PEDOT: PSS: CNT = 110: 1, W/W) was 100.34 Ω/sq.@ 90.1 T%. High transmittance, low sheet resistance, excellent adhesion, and low roughness (3.11 nm) were achieved synchronously. The fabricated OLED demonstrated a low minimum operating voltage (3 V) and could endure high voltage (20 V), at which its luminance reached 2973 cd/m^2^. Thus, the incorporation of CNTs within PEDOT: PSS electrodes has great potential for the improvement of the performance of OLED devices.

## 1. Introduction

Carbon nanotube-based transparent conducting films (TCFs) are widely studied [1], and researchers are trying to apply them to important applications, such as antistatic [2], heating [3], and optoelectronic devices [4]. Commercial optoelectronic products are now widely available, including liquid crystal displays (LCDs) and organic light-emitting diodes (OLEDs), which are in high demand by consumers because of their excellent image quality and contrast, large viewing angle, low consumption of power with high intensity of light, thin structure, and lightweight nature [5]. TCFs are an important part of OLEDs. In these films, transmittance, surface resistance, surface roughness, and other properties determine the performance parameters of OLEDs, such as luminance, external quantum efficiency (EQE), and lifespan. The TCFs of these commercial products are usually made of transparent and conductive-material-doped metal oxides—for instance, indium tin oxide (ITO). Due to the scarcity of indium and the high production costs, these commercialized OLED products are usually expensive. Scientists are trying to replace ITO with alternate materials to reduce the cost of OLED devices. A range of materials have been investigated, including carbon nanotubes (CNTs) [6,7,8,9,10,11], graphene [12,13], graphene oxide (GO) [14,15,16], metallic silver nanowires (AgNWs) [17,18,19], polymeric materials [20,21,22,23,24], and mixtures of these materials [25,26,27,28]. Amongst the alternatives, polymeric materials and CNTs have been selected together, and one prepares TCFs by wet fabrication processes that prepare TCFs quickly with slight fluctuation in sheet resistance and are more suitable for large-scale industrial production conditions. In addition, TCFs produced by polymeric materials usually have high surface quality and robust morphological stability [29,30], which are beneficial for producing OLEDs.

Poly(3,4-ethylenedioxythiophene): poly (styrene sulfonate) (PEDOT: PSS), available as a mixed solution of poly (3,4-ethylenedioxythiophene) (PEDOT) and poly (styrene sulfonate) (PSS), is a widely used conductive polymer with high conductivity and excellent mechanical properties. Nevertheless, there are still many challenges that need to be overcome before PEDOT: PSS can be extensively used in TCFs and OLEDs. They include insufficient conductivity, sensitivity to air, and problems with adhesion to hydrophobic substrates. Pure PEDOT: PSS films usually have very low conductivity, which is not suitable for electrodes, so many techniques have been proposed to enhance the conductivity of PEDOT: PSS films [31]. The approaches include adding organic compounds such as dimethyl sulfoxide, ethylene glycol, and polyethylene glycol, to PEDOT: PSS [32,33]; post-processing PEDOT: PSS films with polar organic compounds such as alcohol or acid [34,35]; or a combination of those methods [36]. Poor operational lifetimes limit the use of PEDOT: PSS in OLEDS [37]. Normally, the enhancement of lifetime is influenced by material stability in different devices [38]. Water is the main component of a PEDOT: PSS dispersion, which will inevitably result in residual water remaining in the spin coated polymer films even after drying. Additionally, PSS is hydrophilic and makes OLEDs susceptible to the influence of water in the air [39]. Wölzl et al. [40] investigated the influence of residual nitrogen, oxygen, and water vapor on the lifetime of OLEDs and found that water vapor introduced a series resistance; the other gases did not influence the electric characteristics of the device. Thus, PEDOT: PSS itself introduces stability problems, which is one of the main problems with degradation in OLEDs [38,39,41]. OLEDs with low stability will form luminance quenchers and carrier traps during operation, which not only lead to a loss of luminance over time along with an increase in operating voltage, but which also may be accumulated due to the continuous electrical stress [42]. Device stability may be improved by optimizing the device structure [43], but the well-known problem for multi-layers is the high fabrication cost [41]. Hydrophobic substrates, such as polyethylene terephthalate (PET) substrate and glass, usually have smooth surfaces, so it is not easy for PEDOT: PSS to adhere to them and form films. Gregori et al. [44] investigated the addition of 1,2-propanediol, ethanol, and diethyleneglycol to PEDOT: PSS so as to increase the adhesion. Kim et al. [45] changed the adhesion of PEDOT: PSS films with sulfuric acid and obtained films with good adhesion via the transfer-printing method. However, the transfer-printing method will bring about the problem of film integrity. Binding agents, such as poly(vinyl alcohol) (PVA), were also used to enhance the adhesion between PEDOT:PSS film and electrode surface [46]; however, the introduction of PVA will affect the electrical properties. Therefore, a major challenge remains: discovering more effective and efficient ways to use PEDOT: PSS in TCFs and OLEDs.

In this study, PEDOT: PSS and CNTs were selected to prepare TCFs using the spin coating method. Differently from the previous treatment of a single reagent, the two-step post-treatment of alcohol and acid was adopted here, so that the properties of the film, including conductivity, could be improved step by step. CNTs have many excellent properties, such as rigidity and conductivity. These properties have been used by many researchers. In addition, carbon is stable in nature. Therefore, based on their own excellent stability, CNTs are considered to enhance the stability of the overall electrodes containing PEDOT: PSS, so as to improve the lifetime of OLED devices. The conductivity, transmittance, roughness, and adhesion of spin-coated films were examined. The surface morphology, structure of films, and mechanism were also analyzed. Furthermore, OLEDs incorporating the films were assessed in terms of device performance with different CNT loadings, via measurements of the luminance, current density, external quantum efficiency, lifetime, and other properties. This research was mainly based on the development of OLEDs based on electrodes containing carbon materials, hoping to inspire the development of other fields [47,48,49,50,51].

## 2. Materials and Methods

### 2.1. Preparation of a CNT/PEDOT:PSS Hybrid Solution

High purity single-walled carbon nanotubes (SWCNTs) (95 wt%, diameter <2 nm, length 5–30 μm) grown by chemical vapor deposition were purchased from Carbon Star High Tech Co. Ltd. (Tianjin, China). Clevios PH1000 PEDOT: PSS aqueous dispersion (Heraeus Ltd., Leverkusen, Germany) with a PEDOT: PSS concentration of 1.0–1.3 wt.% and a weight ratio of PSS to PEDOT of 2.5 was purchased from Poly Light Tech Co. Ltd. (Xi’an, China). Sodium dodecyl benzene sulfonate (SDBS) and Triton X-100 (TX-100) were purchased from Aladdin (Shanghai, China) and were used as received. SWCNTs (1 mg/mL) and SDBS (1 wt.%) were mixed in deionized water and were placed in a bath sonicator for 10 min. After sonicating in a probe sonicator (Bilang Instrument Co., Ltd., Shanghai, China) for 70 min, the solution was centrifuged at 8000 rpm for 15 min followed by decanting 75% suspension into a beaker. Then, 6 mL of the CNT suspension was taken and mixed with distilled water—24, 4, and 0 mL, respectively, corresponding to the CNT concentrations of 0.2, 0.6, and 1.0 mg/mL. Then, 1 mL of PEDOT: PSS solution was filtered through a 0.45 μm polytetrafluoroethylene (PTFE) syringe filter. Following this, CNT solution (0.5 mL) and TX-100 (4 wt.%) were blended to form mixed solutions with CNT concentrations of 0.2, 0.6, and 1.0 mg/mL, respectively. TX-100, a colorless and viscous liquid was used to increase the viscosity of mixed solutions. A sample without CNTs was prepared using deionized water (0.5 mL) instead of CNT solution. Then, each of the four solutions was stirred for 5 min at a speed of 450 rpm to ensure complete mixing.

### 2.2. Fabrication and Post-Treatment of TCFs

**Figure 1:**
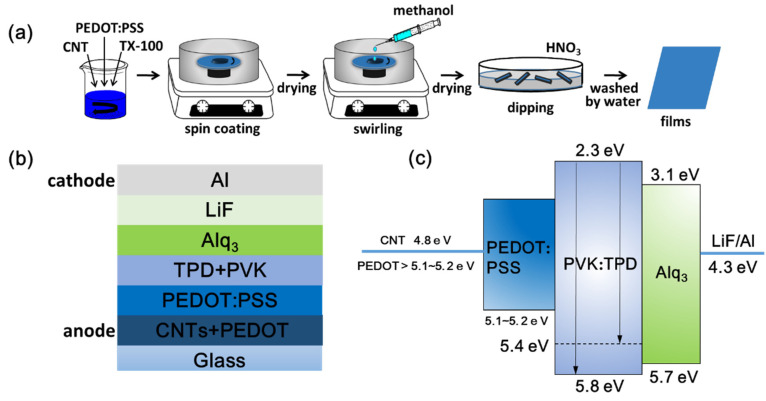
(**a**) A schematic diagram of prepared transparent conductive films. Schematic diagrams of prepared OLEDs: (**b**) structure and (**c**) energy levels.

Glass substrates with an area of 2.0 × 2.0 cm^2^ were cleaned in ethanol for 20 min using a sonicator then blow dried. All processes, including the fabrication and post-treatment of TCFs, were conducted in a glove box, except treatment with nitric acid (Figure 1a). Firstly, 0.1 mL of the mixed solution without CNTs was taken and spin-coated onto glass substrates at a speed of 1000 rpm for 60 s, and the resulting films were thermally annealed at 120 °C for 20 min to remove water. The films obtained were defined as P-TCFs. The same amount of a mixed solution with a CNT concentration of 0.2 mg/mL was used to obtain films followed by thermal annealing at 120 °C for 20 min to remove water, which were defined as CNT/P-TCFs. The CNT/P-TCFs were rinsed with methanol (99.5%, 0.4 mL) at the speed of 2000 rpm for 60 s, twice, followed by thermal annealing treatment at 140 °C for 20 min to remove water and methanol. The corresponding samples are denoted CNT/P-MTCFs. The CNT/P-MTCFs were immersed in acid solution (water: 12 M nitric acid = 1:6 *v/v*) for 40 min, and then rinsed with deionized water to remove residual nitric acid. These films were called CNT/P-NTCFs. These post-processing steps were optimized to achieve best effect. Secondly, the impact of spin-coating speed on the sheet resistance and transmittance of the CNT/P-NTCFs was explored and optimized. Finally, 0.1 mL of the four solutions (with no CNTs and the three CNT concentrations mentioned above) was used to spin-coat films on glass substrates at the optimal speed for 60 s. Then the P-TCFs and CNT/P-TCFs were processed into P-NTCFs and CNT/P-NTCFs after the treatments with methanol (and annealing) and nitric acid. A suitable CNT concentration was obtained by optimizing the values of transmittance and sheet resistance.

### 2.3. Fabrication of OLEDs

The P-NTCFs and CNT/P-NTCFs with different concentrations of CNT solution were made by spin-coating at an optimized speed. The structure and energy-levels of OLEDs are shown in Figure 1b,c [7,12,16,52]. The fabrication steps of the OLEDs were as follows: (1) A copper conductive adhesive used to prepare the anode was stuck on the surfaces of the P-NTCF and CNT/P-NTCF films. (2) In a glove box, PEDOT:PSS solution (150 μL) was dropped on the surface of films and spun for 30 s at a speed of 4000 rpm, and subsequently the films were dried for 15 min at 120 °C for hole buffer layer. This layer cannot only further reduce the surface roughness of the anode, but also reduce the barrier of energy. (3) A mixed liquid including poly(N-vinylcarbazole) (PVK) and N,N’-biphenyl-N,N’-bis(3-methylpheny-l)-1,1’-biphenyl-4,4′-diamine (TPD) was spin coated on the surface twice (200 μL each time), followed by annealing on a hot plate at 50 °C for 20 min for hole transport layer. (4) Templates with three blanks of 3 × 8 mm^2^ were used to cover the films. They were used for evaporating the following layers on the uncovered area, so as to prepare three OLED devices of 3 × 8 mm^2^. (5) The devices were completed by thermal deposition of tris-(8-hydroxyquinolinato)-aluminum (Alq3), LiF, and Al one after another for luminescent layer, electron transport layer and cathode. After the post-treatments described above, the work function of PEDOT: PSS was improved. The final work function of the mixed anodes had a strength between the work functions of CNT and PEDOT: PSS.

### 2.4. Characterization

The sheet resistance of the film was measured using a four-point probe meter (Keithley 2400, Ω-MΩ) (Tektronix technology (China) Co., Ltd., Shanghai, China). The transmittance of the film was measured using an ultraviolet-visible (UV–vis) spectrophotometer at the wavelength of 550 nm, and the transmittance of the glass substrate was subtracted. A field emission-scanning electron microscope (FE-SEM, Hitachi S-4800, Tokyo, Japan) and atomic force microscope (AFM, Bruker, California, USA) were utilized to observe the morphology of the films, and the latter was also used to measure surface roughness. A transmission electron microscope (TEM, TECNAI-20) (Hitachi, Tokyo, Japan) was also used to observe the morphology of the films, prepared by scraping the films off glass substrates using a blade in water with copper grids supporting the films. X-ray photoelectron spectroscopy (XPS, Perkin-Elmer PHI 5600, Al Kα source) (Massachusetts, USA) was used to detect the changes of surface functional groups and elemental content. The performance of OLEDs was tested in a photoelectric tester (SuzhouFstar Scientific InstrumentCo., Ltd., FS-1500GA-OLED) (Suzhou, China).

## 3. Results and Discussion

**Figure 2:**
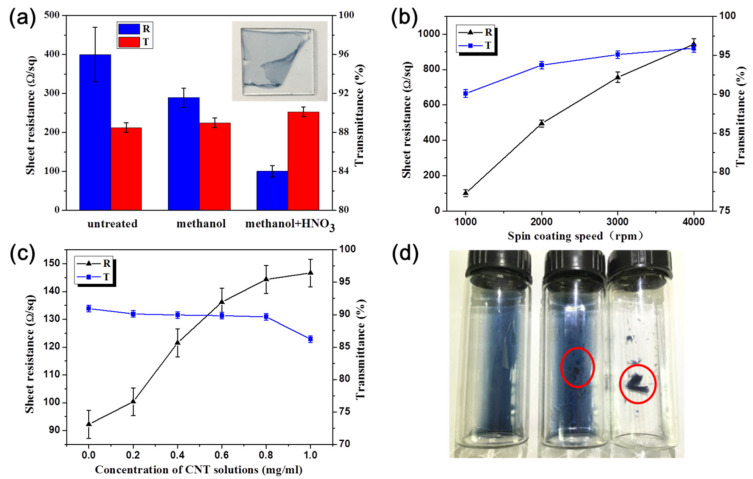
Sheet resistance and transmittance of films prepared under different conditions: (**a**) treatment method (CNT solution: 0.2 mg/mL); (**b**) spinning speed (CNT solution: 0.2 mg/mL); (**c**) concentration of CNT solution; (**d**) PEDOT: PSS hybrid with deionized water, methanol, and nitric acid (from left to right). Red circles in Figure 2d represent different aggregation states of PEDOT:PSS. The inset in Figure 2a shows a film falling off the substrate.

Different factors, including spin-coating speed, carbon nanotube concentration, and treatment methods were considered to discuss the effects on the sheet resistance and transmittance of films (Figure 2). According to Figure 2a, the CNT/P-TCFs have high sheet resistance (400 Ω/sq, with large uncertainty). After rinsing with methanol, the sheet resistance of the CNT/P-MTCFs decreased to about 290 Ω/sq and became more stable than that of the CNT/P-TCFs. The sheet resistance of the CNT/P-NTCFs further decreased to about 100 Ω/sq after the treatment with nitric acid; the smallest fluctuations among the TCFs were present. On the contrary, the transmittance values for these three kinds of TCFs were similar, with values from 88% to 90%. Thus, to reduce sheet resistance, the appropriate method of post-treatment processing is using methanol and nitric acid one after another. Nitric acid alone was not chosen as a control because it caused film debonding from the substrate, as shown in the inset of Figure 2a. Figure 2b shows the effect of spin-coating speed on the sheet resistance and transmittance. The sheet resistance and transmittance all increased at higher spin speeds, while film thickness decreased. Thus, spin coating at a speed of 1000 rpm, leading to low sheet resistance and high transmittance, is optimal. Figure 2c explores the trade-offs between the concentration of the CNT solution and the sheet resistance and transmittance of the CNT/P-NTCFs. The transmittance decreased slightly when the concentration increased from 0.0 to 0.8 mg/mL, and when the concentration increased further, the transmittance decreased significantly to nearly 85%. The sheet resistance increased slowly at the beginning and increased continuously at higher CNT concentrations, before levelling off. This is likely because CNTs did not become the main conductive channel with PEDOT present, as such a network would increase the resistance compared to that of pure PEDOT (92.2 Ω/sq) [53]. As a CNT network develops, it begins to play a role in reducing resistance, so the increasing trend of sheet resistance gradually slows down [53]. As shown in Figure 2d, PEDOT: PSS exhibited different states of aggregation when adding the same volume of deionized water, methanol, or nitric acid. There was no reuniting in the deionized water, and aggregation partially occurred in methanol. Serious reuniting occurred rapidly in nitric acid. PSS was used to disperse PEDOT, and PEDOT aggregated without PSS, as it can [33]. Thus, it is reasonable to assume that methanol and nitric acid can remove PSS from CNT/P-TCFs to various extents, and nitric acid had the better effect. Nitric acid also provides an effect that it can remove SDBS randomly distributed in CNTs [54], but this process needs to take a certain amount of time. On the contrary, the morphology change of PEDOT in nitric acid is very fast, so some SDBS will remain in the films and the higher the CNT content, the more SDBS residue, as indicated by the SEM to be discussed shortly. To test for SDBS residue, XPS measurements were performed, testing for sulfur content (Table 1). Sulfur is present PEDOT: PSS and SDBS, but the differences at a fixed polymer concentration must be due to variations in the SDBS content. The increasing sulfur content indicated more SDBS residue as the CNT concentration increased. Moreover, it can be seen that methanol has no function in removing SDBS. It is reasonable to conclude that the PEDOT films prepared with CNT solutions with high concentrations led to higher sheet resistance than films made from lower-concentration solutions. Fortunately, the CNT conductive network develops with increasing concentration, which offsets some of the increase in sheet resistance caused by SDBS, so the growth rate of sheet resistance slows down (Figure 2c). Finally, through comprehensive considerations of transmittance and sheet resistance, we chose to compare a CNT solution of 0.2 mg/mL with a solution without CNT, to make CNT/P-NTCFs and P-NTCFs, with low sheet resistance and high transmittance. When the concentration of CNT solution was 0.2 mg/mL, the content ratio of PEDOT: PSS to CNT was 110:1. The subsequent discussion of OLED performance is based on samples with that composition. In Table 2, the two groups of performance data are compared with the photoelectric properties of other types of transparent conductive films. The facts show that both groups performed well.

**Figure 3:**
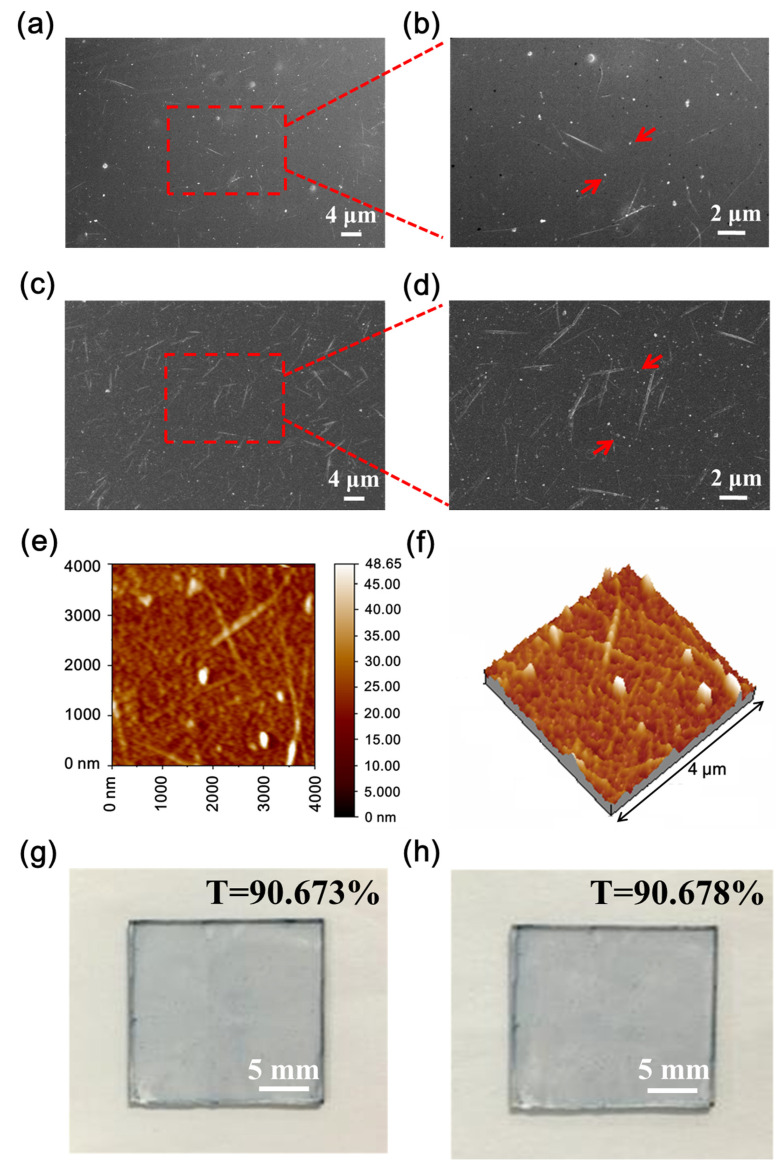
(**a–d**) SEM images of: (**a**) CNT/P-NTCFs prepared from a CNT solution of 0.2 mg/mL and (**b**) an enlarged image of the boxed region in (**a**); (**c**) CNT/P-NTCFs prepared from a CNT solution of 1.0 mg/mL and (**d**) an enlarged image of the central part in (**c**). (**e,f**) The AFM micrograph: (**e**) a topographic image and (**f**) the corresponding three dimensional image of the CNT/P-NTCF. Pictures of CNT/P-NTCF: (**g**) before and (**h**) after the adhesion test.

The structures of the CNT/P-NTCFs prepared from CNT solutions with concentrations 0.2 and 1.0 mg/mL by spin-coating at 1000 rpm were characterized by SEM (Figure 3). The surface morphology of the former was also characterized by AFM (Figure 3e,f). SEM images of the CNT/P-NTCF samples revealed that the CNTs were evenly distributed in the films and the polymer filled the gaps between CNTs (Figure 3a–d). It can be confirmed that some CNT bundles connect with each other and that this is enhanced with higher CNT content, which can be seen more clearly in Figure 3b,d. PEDOT not only forms a compact film but also forms a matrix around the CNTs. On account of the high adhesion between PEDOT and the substrate, the CNTs were well integrated in the films. Furthermore, PEDOT also act as an adhesive between the CNTs, which improves the films’ electrical conductivity [7]. The evenly distributed structure of CNTs is also apparent in the AFM image in Figure 3e. The roughness of TCFs is closely related to the uniform brightness of the OLEDs [57], so it was necessary to test the surface morphology of TCFs. Perfect TCFs will have low roughness. The AFM image in Figure 3e was used to measure the average roughness of the film, which was found to be 3.11 nm, indicating the smooth surface of the whole film. Due to the centrifugal force experienced during spin coating, the CNT bundles were placed in a horizontal direction, as illustrated in Figure 3b,e. It can be assumed that the polymer covers the hollows generated by CNT bundles [7], leading to little overall surface roughness. The above results indicate that the hybrid TCFs are suitable as anode materials for organic optoelectronic devices. Adhesive tape was used to qualitatively explore the adhesion properties of the films. The transmittance changes of pristine films and after peel testing can quantify the degrees of film damage. The relative transmittance value was used to quantitatively characterize the adhesion:

(1) $$f_{R}=\frac{T_{t}-T_{0}}{T_{0}}=\frac{\Delta T}{T_{0}}$$

where $f_{R}$ stands for the relative transmittance; and $T_{t}$ and $T_{0}$ are the transmittances of the CNT/P-NTCFs after and before testing, respectively. Strong adhesion means little difference between the transmittances before and after the trial. That is to say, $T_{n}=T_{0}$ and $f_{R}=0$. On the contrary, when the value of $f_{R}\approx \left(100-T_{0}\right)/T_{0}$, there is no adhesion of the TCFs. As shown in Figure 3g,h, the values of transmittance showed almost no change ($f_{R}=0$), indicating excellent adhesion of CNT/P-NTCFs to the substrate. The qualitative test results of adhesion of different TCFs are listed in Table 3. The results indicate that the presence of PEDOT: PSS led to good adhesion, and the joint action of methanol and nitric acid also improved the adhesion.

**Figure 4:**
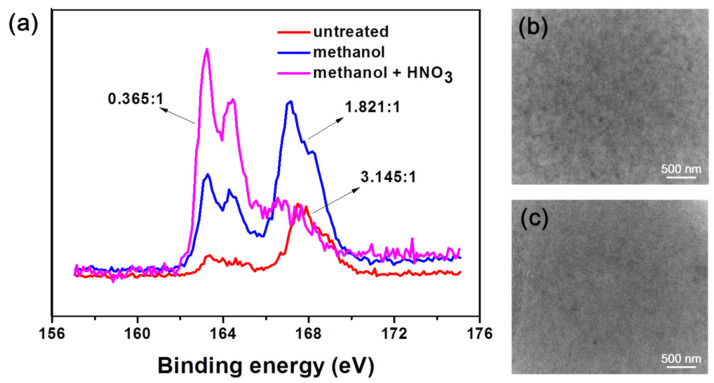
(**a**) XPS spectra of S1s element for PEDOT: PSS films made using different post processing methods (the indicated ratios correspond to PSS: PEDOT, calculated from the spectral area). TEM images of (**b**) P-TCFs and (**c**) P-NTCFs.

XPS was used to investigate the changes of surface functional groups of films fabricated by pure PEDOT: PSS after different treatments (Figure 4a). The TEM images of P-TCFs and P-TCFs treated by methanol plus HNO_3_ are highlighted in Figure 4b,c, respectively. The post treatment of P-TCFs was similar to that of CNT/P-MTCFs and CNT/P-NTCFs. As shown in Figure 4a, the S2p peaks at binding energies of 167.6 and 163.9 eV correspond to the sulfur signals from the sulfonate and thiophene groups of PSS and PEDOT [58], respectively. The calculated ratio of PSS to PEDOT decreased from 3.145:1 to 0.365:1 after the treatment with methanol and nitric acid, signifying that PSS content was markedly reduced, so the sheet resistance of films was lower [36]. This observation suggests that this process, including methanol rising followed by nitric acid treatment, is the most effective method for removing PSS and lower sheet resistance, which is consistent with Figure 2a. The PSS XPS peak showed a small shift (≈0.5 eV) after treatment by methanol, which may have been due to the removal of PSS by methanol, reducing the bond energy [34,59]. The TEM images in Figure 4b,c provide insights into the above process by observing the surface morphology changes of the P-TCFs. The P-TCFs show a granular appearance. After treatment by methanol and nitric acid, the surface morphology became dense and smooth, due to PSS removal and PEDOT rearrangement [34].

**Figure 5:**
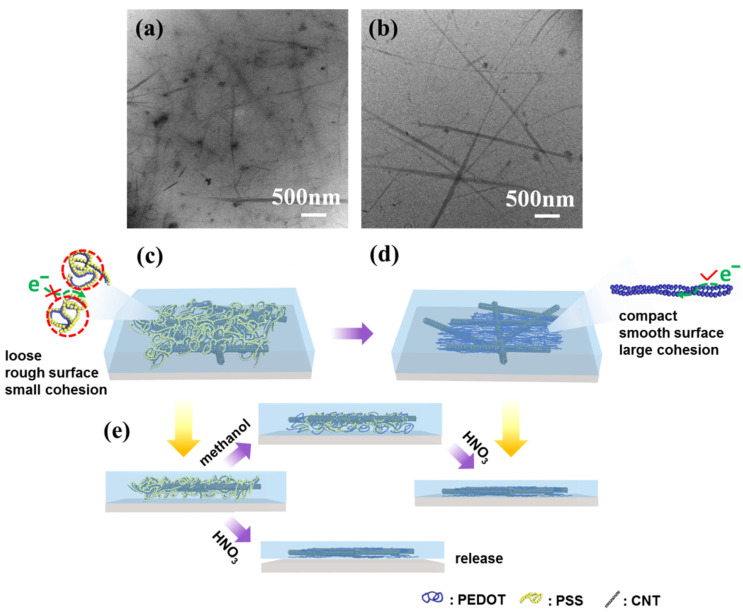
TEM images of (**a**) CNT/P-TCFs and (**b**) CNT/P-NTCFs. The CNT/P-TCFs were prepared at the spin speed of 1000 rpm and CNT solution of 0.2 mg/mL. Corresponding proposed schematic diagrams for the structure of (**c**) CNT/P-TCFs and (**d**) CNT/P-NTCFs. Red lines show spherical PEDOT: PSS chain. (**e**) Schematic of adhesion under different post-treatments.

The morphologies of CNT/P-TCFs and CNT/P-NTCFs were imaged using TEM (Figure 5a,b). A schematic illustration of the structures of CNT/P-TCFs and CNT/P-NTCFs is also shown in Figure 5c,d. The TEM images suggest that PEDOT forms small spherical aggregates of variable size, which are presumably wrapped by PSS, as shown in Figure 5a,c. Owing to the presence of PSS, PEDOT chain connectivity was disrupted, impairing the conductive pathway (and the CNTs also did not form a conductive network). Therefore, the conductivity performance of the CNT/P-TCFs is poor [36]. According to the model proposed in Figure 5c, the CNT/P-TCFs have a loose structure and a rough surface, due to loose interactions between polymer molecules [34]. Moreover, the CNT/P-TCFs will absorb water vapor due to the hydrophilicity of PSS [39]. This can cause the film to debond from the substrate because of low adhesion. As a result of post-treatment, PSS can be effectively removed from the film, which has been proposed to lead to a change in PEDOT chain conformation, from compact to extended, as shown in Figure 5b–d [34]. Then, PEDOT chains aggregated together very densely to form a compact structure, which favors the formation of a conductive pathway without PSS [34]. Moreover, the CNT network surrounded by PEDOT also exhibited better conductivity than before. According to percolation theory, one-dimensional conductive fillers are known to have lower threshold volume fractions for percolation than granular or spherical fillers [60]. It is reasonable to draw the conclusion that given PEDOT an extended conformation can enhance the conductivity, which is supported by the conclusions from Figure 2a,b. The proposed structure of the CNT/P-NTCFs is presented in Figure 5d. The films became very smooth and compact after the treatments with methanol and nitric acid [34]. Meanwhile, the post-treatment had a remarkable effect on increasing the adhesion of CNT/P-TCFs to the glass substrate. Due to the hydrophobicity of PEDOT, the resulting CNT/P-NTCFs can stay dry for a long time in the air [61], thereby preventing release of the film. In terms of adhesion improvement (Figure 5e), TX-100 increased the viscosity of PEDOT: PSS solution so that it could adhere to the surface of glass to form a uniform film. If the film were put directly into concentrated nitric acid, the film would be released from the substrate due to the strong cohesion between the internal molecules of PEDOT [62,63,64]. In the process of methanol treatment, PSS was not substantially removed, so the cohesion is not very strong and the PEDOT chains can partly expand. On the other hand, the film will adhere to the surface of a substrate more closely when methanol is present, due to centrifugal force, which leads to more inter-molecular contacts between PEDOT chains and substrate, and strengthened van der Waals interactions between the film and the glass [45]. Finally, the treatment with concentrated nitric acid further drastically reduced the content of PSS, and this enabled the expansion of the remaining PEDOT chains, leading to more inter-molecular contacts between PEDOT chains and substrate [34]. Then, van der Waals interactions continued to increase, and the adhesion performance of the film was significantly enhanced.

**Figure 6:**
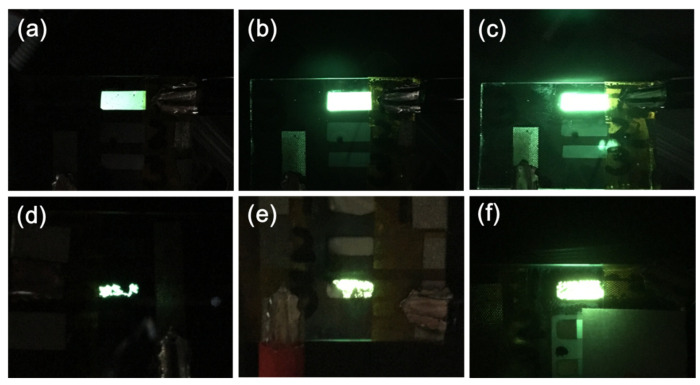
Images of the same OLED (CNT solution: 0.2 mg/mL) under different voltages at time t = 0: (**a**) 4 V, (**b**) 8 V, and (**c**) 20 V. Images of OLEDs with different CNT solution concentrations of (**d**) 0.0 mg/mL, (**e**) 0.2 mg/mL, and (**f**) 1.0 mg/mL driven at 8 V after t = 4 h, under ambient conditions.

Having investigated the morphology and identified conditions for optimal smoothness, transmittance, and conductivity, the performance levels of the CNT/P-NTCFs were next examined. OLEDs fabricated with different CNT contents are shown in Figure 6 at three different voltages. Figure 6a–c show images of the same OLED under 4, 8, and 20 V at time t = 0, in which the electrode was made from a 0.2 mg/mL CNT solution. The whole light area was uniform, and the brightness gradually increased with voltage. In particular, for a voltage of 20 V, the OLED maintained brightness, indicating it could withstand high voltages. Figure 6d–f are images of three different OLEDs with different CNT contents, with the same applied voltage of 8 V after 4 h under ambient conditions. It can be seen that when the content of CNT increased, the ability of the OLED to last and its brightness were also improved. In Figure 6d, the size of the bright area is markedly reduced by 75% and its brightness also decreased significantly after 4 h. When the concentration of CNT was increased to 0.2 mg/mL, the reduction of the bright area with time was slower, about 30%, and its brightness was obviously higher than that of the pure PEDOT: PSS electrode. By increasing the concentration of the CNT solution to 1.0 mg/mL, the size of the bright area decreased slightly, only 5% after 4 h, and the brightness has the least decrement of these three OLEDs in Figure 6d–f. PEDOT: PSS, as a polymer, cannot endure continuous current [42,43], and the brightness loss resulting from use of PEDOT: PSS electrode is larger than that of an electrode based on CNTs [53]. Moreover, a higher CNT content leads to an enhanced CNT network [65,66,67], as shown in Figure 3b,d. Thus, OLEDs containing a pure PEDOT: PSS electrode cannot maintain brightness for a long time. Thus, the CNT network is beneficial in OLEDs electrodes, to enhance stability and prolong lifetime. Through the comprehensive consideration of properties such as sheet resistance, transmittance, and lifetime discussed above, films prepared with 0.2 mg/mL of CNT solution were selected for further study.

**Figure 7:**
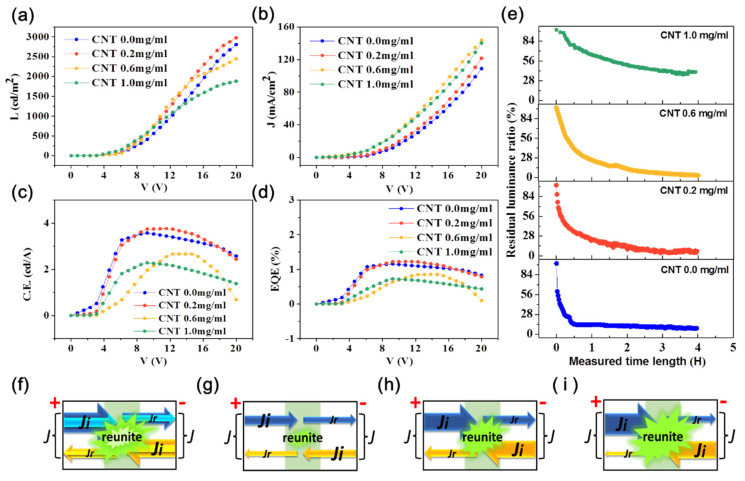
The performances of OLEDs using CNT/P-NTCFs as anodes using CNT solutions with different concentrations (0.0–1.0 mg/mL) and applied with different voltages: (**a**) current density, (**b**) luminance, (**c**) current efficiency, (**d**) external quantum efficiency, and (**e**) lifetime. Schematics of current flow in OLEDs in different states: (**f**) voltage changing from low to high with a certain CNT content, (**g**) low voltage (more CNTs and less CNTs), (**h**) high voltage and more CNTs, (**i**) high voltage and fewer CNTs. (J is the total current of the anode or cathode. The cathode and anode of the same device have the same total current. Marked with $J_{i}$ is the injection limiting current. Marked with $J_{r}$ is the current injected by anode (cathode) but not compounded in the luminescent region and flowing to cathode (anode). The green region represents the light produced by the recombination of electrons and holes.)

The performance of the OLEDs was quantified by a photoelectric tester that had been used to quantify other OLEDs [68]. Measurements of current density, luminance, current density and efficiency, external quantum efficiency, and lifetime are shown in Figure 7. The effect of voltage and concentration of CNT solution were explored, the results being shown in Figure 7a–e. The distribution of currents in the anodes and cathodes of the OLEDs are shown in Figure 7f–i. In Figure 7a, it can be observed that these OLEDs started to function with a low turn-on voltage of 3 V. We combine current density involving the energy levels of the materials in the OLEDs to explain the trend of the measured data. The injection-limited current is given by [69]

(2) $$J_{i}=\;\frac{Ae^{2}E^{2}}{\alpha^{2}\kappa^{2}\Delta }\exp\left(-\frac{2\alpha \Delta^{3/2}}{3eE}\right)$$

and the total current is

(3) $$J=\;J_{i}+\;J_{r}$$

where A,e,α and κ are constants, E is the electric field intensity, Δ is the injection barrier when the electric field intensity is 0, and $J_{r}$ is the current that has not be recombined. The values of luminance all increase with the voltage, and the rates of growth also increase. When the content of CNTs is fixed, Δ is a constant, and $J_{i}$ increases with voltage. The growth of $J_{i}$ means there will be more carriers to combine in the light emitting region in the OLEDs, which can bring higher luminance (Figure 7f). However, the growth trend slows down at high voltages. This phenomenon shows the combination of electrons and holes easily reaches saturation in devices containing more CNTs. When the voltage is fixed, the E is a constant and the values of $J_{i}$ will reduce with increasing CNT content bringing reduced Δ. According to Figure 1c, the energy barrier at the cathode is larger than that at the anode, so it is harder for electrons to enter the OLEDs than holes. As limited electron flow occurs at low voltage, few photons are produced by the combination of electrons and holes, meaning CNT content has little effect on OLED performance (Figure 7g). At high voltage, the numbers of holes and electrons both increase. OLEDs with higher CNT content inject fewer holes than those with lower CNT content within a given time due to the high energy barrier (under conditions with fixed number of electrons). This leads to a reduction of luminance with increasing CNT content (Figure 7h,i). The absorption of photons by carbon nanotubes also partly contributes to the reduction of luminance [1,70]. However, the performance of OLEDs prepared from the 0.2 mg/mL CNT solution was better than that of the OLEDs without CNTs, especially at high voltages. This reflects the fact that the addition of CNTs enhances the ability to endure high voltages, so the OLED can perform better. The highest value of luminance was 2973 cd/m^2^ with an emission peak at approximately 530 nm for the OLED fabricated by the CNT solution of 0.2 mg/mL at voltage of 20 V. Its performance is superior to that of other OLED devices of the same material [7,31].

Figure 7b shows the current density changing with the applied voltage. The values of current density all increased with voltage, especially when the content of CNTs was high. The current density of OLEDs with high CNT content at the same voltage was higher and increased faster with voltage than that of OLEDs without CNTs. These phenomena are also explained through an analysis of current density, considering the energy levels of the materials in the OLEDs. When the concentration of the carbon nanotube solution is fixed, Δ will be a constant, and the value of $J_{i}$ will increase with E (and so voltage). At high voltage, even if the carriers involved in recombination increase, the growth rate of carrier injection is faster than that of recombination, so the overall trend is for the current to grow (Figure 7f). When the value of voltage is fixed, E will be constant. According to Figure 1c, the energy difference between anode and hole injection layer increases with the concentration of the carbon nanotube solution, and the value of $J_{i}$ will reduce. At low voltages, the values of J for different CNT concentrations do not have large differences, because the values of $J_{i}$ and $J_{r}$ are all very small, which results from low voltages (Figure 7g). At high voltages, J of the cathode, composed of $J_{r}$ from anode and $J_{i}$ of cathode, is the focus here, because the current of the anode is the same as that of the cathode. At the anode, $J_{i}$ at low CNT concentrations will be larger than at high concentrations, which means that the high barrier will hinder carrier injection and photon conversion. According to Figure 7a, the luminance of OLEDs having low CNT content increases rapidly at high voltage, but due to the relatively small photon efficiency [71], more holes need to be consumed, resulting in the fewer residual holes than at high CNT content ($J_{i}$ values for the cathodes are the same). Finally, the whole trend of J is that OLEDs containing more CNTs have a high current (Figure 7h,i).

Figure 7c shows data for current efficiency (C.E.) versus voltage. The value of C.E. can be calculated by the following formula:

(4) $$\mu_{L}=\;\frac{AL}{I_{OLED}}$$

where $\mu_{L}$ is the current efficiency, A is the effective area, L is luminance, and $I_{OLED}$ is the current. As shown in Figure 7c, the maxima in these curves reduce with increasing CNT content, except for 0.2 mg/mL. The reason is that luminance gradually reduces with voltage and $I_{OLED}$ gradually increases, both resulting in the reduction of $\mu_{L}$. The better performance of the OLED prepared from the 0.2 mg/mL CNT solution at high voltage and the ability to endure high voltages were due to the presence of CNTs. The highest value of C.E. in Figure 7c was 3.76 cd/A. Han et al. [72] fabricated OLEDs by modifying the graphene anode, which could reach 30 cd/A, but this value could only be maintained when the voltage was about 2.5 V, and the performance decreased badly at higher voltages, indicating that the OLEDs were not very stable. In this work, when the content of CNTs was low, the corresponding C.E. values were relatively high and relatively stable for voltages between 6 to 18 V, especially when the concentration of CNT solution was 0.2 mg/mL. The C.E. values do not vary too much over a wide range of voltage for OLED operability.

External quantum efficiency (EQE) is the ratio of the number of photons produced by OLEDs to the number of electrons injected into OLEDs. With the increase of CNT content, the number of electrons injected into OLEDs is a constant, but the number of photons is reduced, so the EQE by its nature shows a decay as a function of time. What is surprising is that the OLED fabricated by the CNT solution with 0.2 mg/mL had a better performance than the pure electrode, which means that the addition of CNTs is beneficial to maintaining performance. According to Figure 7d, the highest value of EQE was 1.23%.

Lifetime is a vital factor for OLEDs, and the test for the lifetime is usually carried out under the condition that OLED devices are packaged, which aims to eliminate the effects of water and oxygen in the air on the device, but the ability to maintain lifetime of the OLED itself is also critical to the whole device. We tested the lifetime of OLEDs in ambient conditions without encapsulation, and the result is shown in Figure 7e. Every OLED device was lit for 4 h at a voltage of 8 V, and the residual luminance ratio was measured during this period to evaluate the performances of the OLEDs, including durability, stability, and lifetime. The definition of the percentage residual luminance is

(5) $$L_{r}=L_{t}/L_{i}\times 100\%,$$

where $L_{r}$ stands for percentage residual luminance, and $L_{t}$ and $L_{i}$ are luminance at time t and initial luminance, respectively. According to Figure 7e, the reduction of the percentage of residual luminance decreases with increasing CNT concentration. The percentages of residual luminance corresponding to the concentrations of CNT solution of 0.0, 0.2, 0.6, and 1.0 mg/mL at half an hour were 14.2%, 34.2%, 40.93%, and 76.75%, respectively.

## 4. Conclusions

Hybrid PEDOT: PSS/CNT films were prepared by spin coating, and were used as the anodes for OLEDs. Methanol rising was used to remove PSS to improve surface smoothness and enhance adhesion performance. Nitric acid was used to remove PSS efficiently, which improved PEDOT chain connectivity, contributing to reduced sheet resistance, lower roughness, and enhanced adhesion. The optimized concentration for the CNT solution and speed for spin coating were found to be 0.2 mg/mL and 1000 rpm, respectively. Successive treatment with methanol and nitric acid led to a film with a relatively low sheet resistance of 100.34 Ω/sq and transmittance of 90.1%. This film also had the advantages of strong adhesion and low surface roughness (3.11 nm) which are beneficial for OLEDs to inject carriers to the next layer so as to obtain uniform brightness. The OLEDs were shown to have low turn-on voltage (3 V) and had the highest luminance of 2973 cd/m^2^ with an emission peak at approximately 530 nm when the voltage was 20 V, and also showed the ability to withstand high voltages. The addition of CNTs also enhanced the stability and prolonged the lifetime of the OLEDs. This study paves the way for the development of OLEDs based on electrodes containing carbon materials for advanced energy and related applications.

## Figures and Tables

**Table 1:** Content of S (wt %) in CNT/P-NTCFs prepared by CNT solutions of different concentrations.

Concentrations of CNT Solutions (mg/mL)	0.0	0.2	1.0
**Content of Element S (%)**	5.47 ± 0.2	6.45 ± 0.2	8.28 ± 0.2

**Table 2:** A comparison of the electric and optical properties of different types of transparent conductive films.

Materials	Sheet Resistance (Ω/sq)	Transmittance (T%)	Ref.
SWCNTs	128	90	[8]
SWCNTs	124	80	[11]
SWCNTs	86	80	[54]
PEDOT: PSS	113	91	[32]
AgNWs	86	80	[18]
reduced GO	642	80	[15]
PEDOT: PSS	92.2	92	Our work
SWCNTs, PEDOT: PSS	100.34	90.1	Our work
SWCNTs, PEDOT: PSS	70.6	81	[7]
CNTs, PEDOT: PSS	173	55.2	[23]
CNTs, PEDOT: PSS	95.15	87.72	[31]
CNTs, PEDOT: PSS	89.1	91	[55]
SWCNTs, AgNWs	29.2	80	[17]
CNTs, GO	146	86	[25]
PEDOT: PSS, GO	84	87	[56]

**Table 3:** Adhesion testing of different TCFs.

Type of TCFs	Type of Post-Treatment	
	Methanol	Methanol+HNO_3_
CNT	Complete removal	Complete removal
CNT + TX-100	Complete removal	Complete removal
CNT + TX-100 + PEDOT: PSS	Less removal	No removal

## Data Availability

Data are contained within this article.
